# Antifungal Chitosan Nanocomposites—A New Perspective for Extending Food Storage

**DOI:** 10.3390/ijms252313186

**Published:** 2024-12-08

**Authors:** Natalia Wrońska, Aleksandra Felczak, Katarzyna Niedziałkowska, Marta Kędzierska, Maria Bryszewska, Mohamed Amine Benzaouia, Abdelkrim El Kadib, Katarzyna Miłowska, Katarzyna Lisowska

**Affiliations:** 1 Department of Industrial Microbiology and Biotechnology, Faculty of Biology and Environmental Protection, University of Lodz, 12/16 Banacha Street, 90-236 Lodz, Poland; aleksandra.felczak@biol.uni.lodz.pl (A.F.); katarzyna.niedzialkowska@biol.uni.lodz.pl (K.N.); katarzyna.lisowska@biol.uni.lodz.pl (K.L.); 2 Department of General Biophysics, Faculty of Biology and Environmental Protection, University of Lodz, 141/143 Pomorska Street, 90-236 Lodz, Poland; marta.kedzierska@umed.lodz.pl (M.K.); maria.bryszewska@biol.uni.lodz.pl (M.B.); katarzyna.milowska@biol.uni.lodz.pl (K.M.); 3 Engineering Division, Euromed Research Center, Euro-Med University of Fes (UEMF), Route de Meknes, Rond-Point de Bensouda, Fès 30070, Morocco; m.benzaouia@ueuromed.org (M.A.B.); a.elkadib@ueuromed.org (A.E.K.)

**Keywords:** antifungal chitosan films, zinc oxide, genotoxicity, food spoilage prevention, food packaging materials

## Abstract

Chitosan, a biopolymer derived from chitin, exhibits significant antifungal properties, making it a valuable compound for various applications in agriculture food preservation, and biomedicine. The present study aimed to assess the antifungal properties of chitosan-modified films using sol–gel derivatives (CS:ZnO) or graphene-filled chitosan, (CS:GO and CS:rGO) against two strains of fungi that are the most common cause of food spoilage: *Aspergillus flavus* ATCC 9643 and *Penicillium expansum* DSM 1282. The results indicate important differences in the antifungal activity of native chitosan films and zinc oxide-modified chitosan films. CS:ZnO nanocomposites (2:1 and 5:1) completely inhibited spore germination of the two tested fungal strains. Furthermore, a decrease in spore viability was observed after exposure to CS:Zn films. Significant differences in the permeability of cell envelopes were observed in the *A. flavus*. Moreover, the genotoxicity of the materials against two cell lines, human BJ fibroblasts and human KERTr keratinocytes, was investigated. Our studies showed that the tested nanocomposites did not exhibit genotoxicity towards human skin fibroblasts, and significant damage in the DNA of keratinocytes treated with CS:ZnO composites. Nanocomposites based on chitosan may help reduce synthetic fungicides and contribute to sustainable food production and food preservation practices.

## 1. Introduction

The popularity of eco-friendly packaging materials is constantly growing due to the need to reduce the negative impact of plastic pollution on the environment. In recent years, chitosan has become a biomaterial of increasing interest in the biomedical (wound dressings, drug delivery systems, and tissue engineering scaffolds) and food industries (packaging material). Chitosan can be modified to obtain good biological activity thanks to the presence of functional groups.

Chitosan is a biodegradable polysaccharide with an excellent film-forming ability [1,2]. It is derived from chitin, which is found in the exoskeletons of crustaceans (e.g., shrimps and crabs) and in the cell walls of fungi [3]. Crustacean shells are the main source of chitosan. It is a waste material, easily available, and does not generate high processing costs [4]. Biodegradability and biocompatibility are common properties of a wide group of biopolymers. The universal use of chitosan is mainly due to the presence of amino groups in the polymer backbone, giving it several valuable properties, including catalytic and antibacterial effects and the ability to chelate metals [5,6,7].

Chitosan films have gained significant attention due to their unique properties such as biocompatibility [8], antimicrobial activity [9], flexibility, and biodegradability [10,11]. Moreover, chitosan film has excellent barrier properties, making it suitable for food packaging applications to prevent spoilage and maintain product freshness [12,13]. Chitosan has been proven to promote tissue regeneration and wound healing due to its hemostatic, mucoadhesive, and biostimulatory properties [14]. Another important aspect is its mechanical strength, which can be controlled by varying the chitosan concentration, molecular weight, and crosslinking agents [15,16]. Our previous work demonstrated that the addition of graphene or ZnO allowed us to obtain stable chitosan nanocomposites with excellent mechanical properties, thermal stability, and antibacterial activity [17,18]. The addition of the abovementioned compounds increases the stability of the film via creating strong interactions with the chitosan matrix [19,20]. Importantly, the combination of ZnO or graphene with a chitosan matrix does not significantly affect the biodegradability of these materials [11].

The antifungal activity of chitosan is attributed to its ability to disrupt the cell walls and membranes of fungi. This polymer can induce morphological changes, structural alterations, and molecular disorganizations in the fungal cells [21,22]. The functional chitosan amino groups react with the negatively charged components of fungal cell walls, such as chitin and glucans, causing an increase in the permeability of the fungal cell membrane [23]. Moreover, this biopolymer might have an effect on the synthesis of fungal enzymes essential for fungal growth and metabolism [24]. The antifungal activity of chitosan is influenced by its degree of deacetylation and molecular weight and the pH of the surrounding environment. Chitosan is most effective as an antifungal agent under acidic conditions, where it carries a positive charge, facilitating interactions with negatively charged fungal cell walls [25].

Chitosan nanocomposites can be used as a bioactive coating for food packaging materials to extend the shelf life of perishable products by inhibiting microorganism’s growth and spoilage. In our previous work, we succeeded in entrapping a variety of metal oxide nanoparticles inside the films, including binary and ternary mixed oxide [17]. Good quality, transparent, and flexible micrometer-thick films highly suitable for coating and packaging applications were straightforwardly obtained. Some of these functional films exhibited excellent antibacterial effects against bacteria causing food spoilage [26]. These interesting results prompted us to further assess the antifungal properties of the selected chitosan composites against *Aspergillus flavus* ATCC 9643 and *Penicillium expansum* DSM 1282. The fungi from *Aspergillus* and *Penicillium* genera are the most common cause of food contamination. Importantly, the contamination of vegetables and fruit may occur at various stages of production, from harvesting through processing to storage [27,28]. These fungi can produce various mycotoxins that are hazardous to animals and humans. Moreover, strains of the *Aspergillus* genus cause many human diseases, from wound infections to invasive fungal rhinosinusitis and endophthalmitis, as well as pulmonary *aspergillosis* [29].

Regulatory agencies and safety organizations, such as the European Food Safety Authority (EFSA) and the U.S. Food and Drug Administration (FDA), require comprehensive safety evaluations before a material can be approved for food contact. An important aspect of testing the toxicity of composites with potential use as food storage films is determining their genotoxicity. The effect of chitosan composites on the DNA of skin cells is an extremely important stage of in vitro research; therefore, the genotoxicity of chitosan films was tested against two cell lines, human BJ fibroblasts, and human KERTr keratinocytes.

The present study aimed to assess the antifungal properties of films based on chitosan with supplemental compounds—metal oxide (ZnO) and graphene derivatives including graphene oxide GO and reduced graphene oxide rGO—against *A. flavus* ATCC 9643 and *P. expansum* DSM 1282. Furthermore, the genotoxicity of the tested materials was assessed to examine the safety of their use in the packaging industry.

## 2. Results

### 2.1. Film Characterization

Detailed characterization of the chitosan films used in the presented work is available in our previous works [17,18,26].

### 2.2. Antifungal Activity

**Figure 1:**
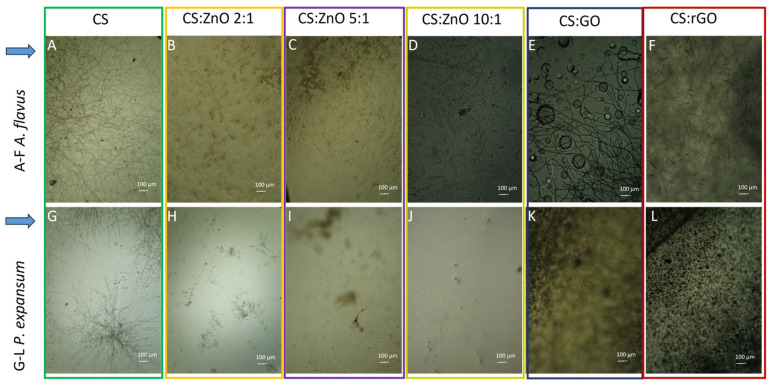
Optical microscopy images of chitosan, chitosan–metal oxide, and chitosan–graphene oxide films after 24 h incubation with fungal spores ((**A**–**F**)—*Aspergillus flavus*; (**G**–**L**)—*Penicillium expansum*). (**A**,**G**)—CS; (**B**,**H**)—CS:ZnO 2:1; (**C**,**I**)—CS:ZnO 5:1; (**D**,**J**)—CS:ZnO 10:1; (**E**,**K**)—CS:GO; (**F**,**L**)—CS:rGO. Magnification: 400×.

The first stage of the research, which allowed the selection of nanocomposites with the highest antifungal potential, is shown in Figure 1. Both the tested fungal strains *A. flavus* and *P. expansum* showed excellent growth on pure chitosan film. Developed sporangia were also noticeable. In the case of the CS:Zn 2:1 film, no growth of *A. flavus* was observed, while in the case of CS:Zn 5:1, single-lysed hyphae were visible. Single germinating spores were recorded when the CS:Zn ratio was 10:1. Mycelium growth and numerous sporangia were observed on chitosan films modified by graphene oxide (Figure 1E). In the case of the *P. expansum* incubated on the selected chitosan films, films with zinc also showed excellent antifungal properties. Microscopic analyses showed ungerminated spores and single germinating ones. Nanocomposites with graphene did not present antifungal potential against the *Penicillium* strain. Zinc-modified chitosan films were selected for further research.

**Figure 2:**
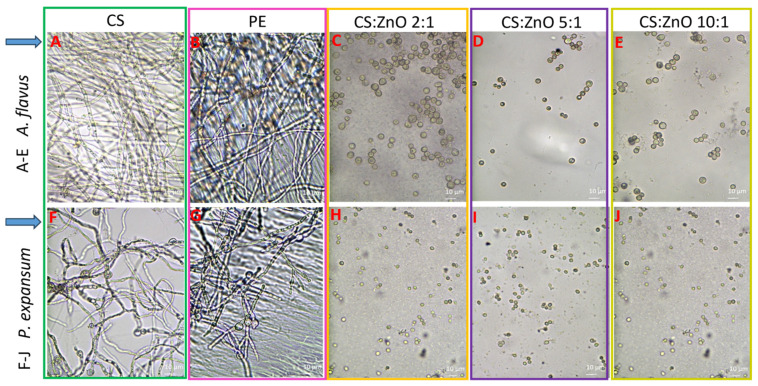
Optical microscopy images of chitosan, chitosan–metal oxide, and chitosan–graphene oxide films after 24 h incubation with fungal spores ((**A**–**E**)—*Aspergillus flavus*; (**F**–**J**)—*Penicillium expansum*). (**A**,**F**)—CS; (**B**,**G**)—polyethylene foil; (**C**,**H**)—CS:ZnO 2:1; (**D**,**I**)—CS:ZnO 5:1; (**E**,**J**)—CS:ZnO 10:1. Magnification: 400×.

In the next stage of the research, to confirm the antifungal effect of the selected modified chitosan films, the amount of spore suspension applied to the tested nanocomposites was increased threefold. In addition, commercially available polyethylene foil was included in the research. The results are shown in Figure 2. In the case of both tested fungal strains, abundant mycelial growth was observed on the pure chitosan film and the polyethylene film (Figure 2A,B—*A. flavus*; F,G—*P. expansum*). The chitosan composites modified with zinc oxide in the CS:Zn ratio of 2:1, 5:1, and 10:1 showed strong fungistatic properties, and the complete inhibition of fungal spore germination was noted in the case of both *A. flavus* and *P. expansum.*

### 2.3. Evaluation of the Mechanisms of Antifungal Action of Chitosan Films

Long-lasting viable fungal spores are an important aspect of the formation and spread of fungi causing food spoilage. At this stage of our research, we used a system using Alamar Blue (resazurin dye; 7-hydroxy-3H-phenoxazin-3-one 10-oxide) to assess the viability of fungal strains using *A. flavus* and *P. expansum*. The assay depends on the metabolic activity of viable fungal strains converting the dark blue of resazurin (maximum absorbance 605 nm) into the pink color of resorufin (maximum absorbance 573 nm). The control was a pure chitosan film with 100% spore viability.

**Figure 3:**
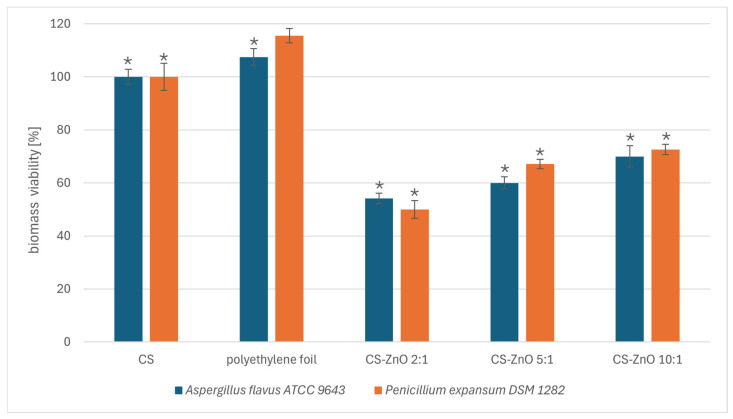
The viability of *A. flavus* and *P. expansum* biomass after 48 h of incubation on the selected chitosan films. The data represent the mean ± SD of three different experiments performed in triplicate. The value of the control group (CS) was set as 100%. Statistical significance is marked in relation to the chitosan nanocomposite (CS) (*n* = 3, * *p* < 0.05).

Across all the test nanocomposite materials, the highest percentage of viable fungal biomass after 48 h of incubation was from the polyethylene film. The viability measurement of fungal biomass showed significant differences after incubation on chitosan nanocomposites supplemented with zinc in the ratio of CS:Zn equal to 2:1, 5:1, and 10:1 (Figure 3). *A. flavus* and *P*. *expansum* viability decreased by approximately 30–50% and 30–40%, respectively, compared to the pure chitosan film. These results are consistent with those determining the fungistatic potential of the chitosan nanocomposites, where the CS:Zn 2:1 and CS:Zn 5:1 films completely inhibited spore germination (Figure 2).

**Figure 4:**
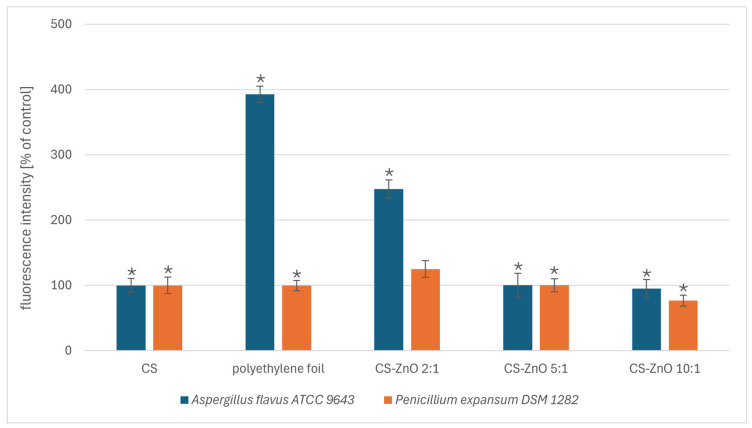
The effect of the tested nanocomposites and PE foil on the membrane permeabilization of *A. flavus* ATCC 9643 and *P. expansum* DSM 1282. The data represent the mean ± SD of three different experiments performed in triplicate. The value of the control group (CS) was set as 100%. Statistical significance is marked in relation to the chitosan nanocomposite (CS) (*n* = 3, * *p* < 0.05).

To investigate membrane permeabilization in fungi exposed to the tested nanocomposites, we used an assay based on the uptake of SYTOX Green. Significant differences in the permeability of cell envelopes were observed for the *A. flavus* strain (Figure 4). In this case, significant changes were caused by polyethylene foil and CS:ZnO 2:1. It should be noted that a longer incubation time caused the dye to interact with the tested nanocomposites, making it difficult to read the results. Therefore, it was decided to apply a limited incubation time.

### 2.4. Genotoxicity of Chitosan Films

**Figure 5:**
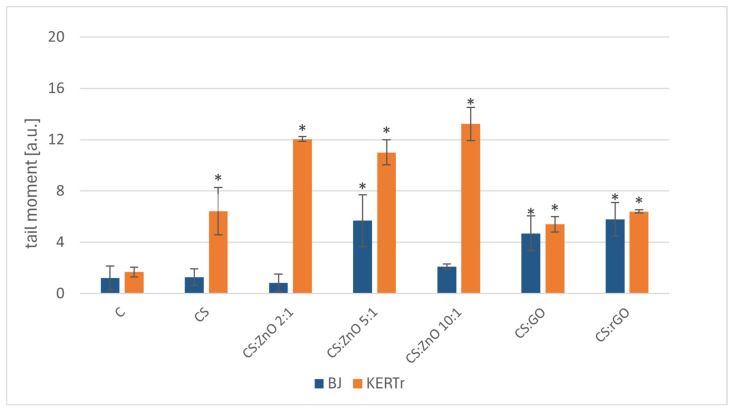
The genotoxicity of tested nanocomposites to BJ and KERTr cells incubated for 24 h (*n* = 6. * *p* < 0.05).

A comet assay can identify single- and double-strand DNA damages, as well as any chemical and enzymatic modifications that may lead to DNA or chromatid breaks. Figure 5 shows the tail moment as a percentage equivalent to DNA damage. After 24 h of incubation, the tail percentage for BJ was a maximum of 6%, and for KERTr, approximately 12–13%. For fibroblasts, a statistically significant increase in tail moment compared to the control was observed for CS:ZnO 5:1, CS:GO, and CS:rGO. In the case of keratinocytes, the strongest DNA-damaging effect was demonstrated by the CS:ZnO composites; however, no differences were observed between the amounts of ZnO in the composite. A significant increase in DNA damage was also observed for CS, CS:GO, and CS:rGO, but these values were similar to those obtained for BJ cells and did not exceed 6.5%.

## 3. Discussion

The increase in environmental pollution as a result of plastic waste, including microplastics, has motivated researchers to test films based on biopolymers to introduce ecological packaging materials. Polymer matrices that are based on biopolymers have become popular due to their film-forming properties, biodegradability, biocompatibility, low toxicity, and antimicrobial potential. Chitosan, a biopolymer derived from chitin, has emerged as a promising material for the development of sustainable packaging solutions. In the presented work, the implementation of advanced functional coatings based on chitosan has been explored for the design of antifungal films. We tested the antifungal effects of CS:ZnO in different ratios and GO and rGO chitosan films against two strains of filamentous fungi: *Aspergillus flavus* and *Penicillium expansum*. First of all, the experimental results indicate that the zinc-modified chitosan nanocomposites had the strongest fungistatic effect. Surprisingly, chitosan–graphene nanocomposites did not show antifungal potential against the two tested filamentous fungi. Mycelium growth and numerous sporangia were observed. Even with a threefold increase in the amount of spore suspension applied to the tested nanocomposites, the antibacterial potential of the zinc-modified films was maintained. Huang et al. (2022) investigated the effect of GO in chitosan/alginate-based buccal foams on the antifungal activity, concluding that the addition of 1% wt% GO did not effect the antifungal effect on clotrimazole-loaded foams [30]. Another study showed that the combination of GO and CS resulted in a significant synergistic inhibitory effect on the mycelial growth of *F. graminearum* compared with single GO or CS [31]. There are limited literature data on the antifungal properties of the combination of ZnO and chitosan against food spoilage pathogens. The antifungal activity of coating films made from chitosan combined with zinc oxide nanoparticles (ZNPs) and sandalwood essential oil (SEO) was studied [32]. It was found that the addition of 0.025% ZNP and 0.5% SEO to 0.8% chitosan coating solution exhibited an excellent effect on the inhibition of both the mycelial growth and spore germination of *Penicillium italicum*. Park et al. (2006) showed that the use of chitosan alone was not enough to restrain the growth of *Rhizopus* sp., and thus, it was necessary to include potassium sorbate to obtain total inhibition [33]. In our studies, no antifungal effect of pure chitosan film was observed. In the case of both tested fungal strains, abundant mycelial growth was observed. The antifungal action against *Candida albicans* has been described for solutions of low-molecular-weight chitosan [34]. Marin et al. (2013) showed the growth-inhibitory effect of vanillin–imino-chitosan films on the *C. albicans* fungus strain [35]. Other works described the antifungal potential of chitosan-based films with 2-aminothiopene derivatives against *Candida* species: *C. albicans*, *C. tropicalis*, and *C. parapsilosis* [36]. Kraisit et al. (2022) suggested the usefulness of chitosan films coating fluconazole-loaded solid lipid nanoparticles in the treatment of candidosis via the buccal mucosa [37]. Different results for the chitosan solution were obtained by Sebti et al. (2005). They showed 100% *A. niger* spore inhibition using chitosan solutions (0.1 and 1% x/v) [23]. On the other hand, Zhiani et al. (2009) demonstrated the antifungal activity against *A. alternaria* of both films and solutions based on chitosan and surfactant, although solutions exhibited higher effectiveness [38]. They also proved that chitosan films were more effective against *A. niger*. Murillo et al. (2023) reported antifungal activity against *Pleurotus ostreatus* by a combination of chitosan and silver nanoparticles, using the dual fabrication process of electrospinning and dip-coating techniques [39]. In the presented work, we demonstrated, for the first time, the high antifungal activity of chitosan films supplemented with ZnO against filamentous fungi (*A. flavus* and *P. expansum*), which are common food pathogens.

Fungal spores are an important aspect of the spread of fungi causing food spoilage. Therefore, our work demonstrated the influence of zinc-modified chitosan films (CS:Zn 2:1 and 5:1) on a significant reduction in the germination of *A. flavus* and *P. expansum* spores and their viability. Linklater et al. (2023) tested the viability of *Aspergillus brasiliensis* spores in contact with hydrophobic and hydrophilic nanopillar silicon surfaces [40]. The result showed that the spore metabolic activity of conidia attached to the nano silicon surfaces was reduced after 72 h incubation.

One of the suggested mechanisms of action of chitosan on filamentous fungi is the disruption of membrane permeability [23,41]. This disruption can lead to the leakage of intracellular components, loss of ions and metabolites, and ultimately cell death. Sensitive fungi showed energy-dependent plasma membrane permeabilization by chitosan [42]. Moreover, the plasma membranes of chitosan-sensitive fungi were shown to have more polyunsaturated fatty acids than chitosan-resistant fungi, suggesting that their permeabilization by chitosan may be dependent on membrane fluidity [43]. In our studies, significant disturbances in the permeability of the fungal membrane were visible only for the *A. flavus* strain during incubation on the CS:ZnO 2:1 film and polyethylene foil. Mesas et al. (2021) analyzed the cell membrane permeability of *Fusarium eumartii* spores incubated with N-methylene phosphonic chitosan by using SYTOX Green [44]. An increase in membrane permeability was observed by 22% after 1 h and by 65% after 4 h of incubation time.

It is crucial to examine the performance of the materials being considered for potential use in food storage, with a particular focus on assessing their genotoxic properties. To address this, the comet test was utilized to determine whether the composites cause single- or double-strand DNA breaks. The genotoxicity of substances can arise from their direct interactions with DNA or from an indirect reaction triggered including the release of toxic ions/oxides from the tested materials or oxidative stress [45,46,47]. Our studies have shown that the tested nanocomposites did not show genotoxicity towards human skin fibroblasts. A statistically significant increase in the value of the tested parameter (tail moment) was observed, but these changes did not exceed 6.5%. However, keratinocytes were more sensitive to the effects of the tested composites, especially those containing zinc oxide, which is the main factor responsible for the increase in the genotoxicity of these materials. The results were also confirmed in other earlier studies, where alginate composites containing zinc oxides showed higher genotoxicity towards human skin keratinocytes than alginate composites [48]. Previous studies carried out mainly with animals have also shown the genotoxicity of zinc oxide nanoparticles. The direct mechanisms of metal oxide genotoxicity (including zinc) are based on DNA strand breaks [49,50,51]. The genotoxic effects of ZnO were also investigated by Ng et al. (2017) [52]. They observed DNA oxidation in MRC5 cells (human lung fibroblasts) exposed to ZnO NPs. An increase in oxidative DNA damage products and DNA strand breaks confirmed that ZnO NPs induce DNA damage. A similar DNA-damaging potential of ZnO NPs was also observed in Sharma et al.’s (2009) studies in the human epidermal cell line (A431) [53].

Chitosan-modified films have a wide range of potential applications, particularly in the food, medicine, and environmental protection industries. Chitosan films supplemented with zinc could be employed as innovative food packaging materials, leveraging the antimicrobial properties of both components. These films could extend the shelf life of perishable products such as vegetables, fruits, and meats by reducing microbial contamination. Moreover, the tested materials could lower the need for synthetic preservatives in food. Considering the practical use of chitosan nanocomposites, possible limitations should be mentioned. Chitosan films tend to be brittle, which can affect their durability during transport. In addition, they usually have relatively high oxygen and water vapor permeabilities, which reduces their ability to maintain the shelf life of moisture-sensitive food products [13]. Therefore, modifying the chitosan coating by adding some components, e.g., metal oxides, can change the mechanical strength and flexibility of the films. Considering that the combination of zinc with chitosan enhanced both the antifungal activity of the coatings and their mechanical properties, this combination is the best candidate for future research. With the continuous advancement in material science and sustainable production techniques, zinc–chitosan films can play a key role in solving challenges in food security and environmental sustainability.

## 4. Materials and Methods

### 4.1. Materials

Chitosan of medium molecular weight and 85% deacetylation degree, and zinc acetate (Zn(OAc)2) were purchased from Sigma-Aldrich (Hamburg, Germany). Graphite flakes, potassium permanganate, sodium nitrate, sulfuric acid, hydrochloric acid, hydrazine, hydrogen peroxide, phosphoryl chloride, bis-trimethylsilylamine, ethanol, tetrahydrofuran, and acetic acid were obtained from Across and Sigma-Aldrich (Darmstadt, Germany). SYTOX Green Nucleic Acid Stain and Alamar BlueTM were purchased from ThermoFischer Scientific (Budapest, Hungary).

The human skin line fibroblast BJ (CRL-2522) and keratinocyte CCD 1102 KERTr (CRL-2310) cell line were purchased from American Type Culture Collection ATCC^®^ (Manassas, VA, USA). Keratinocyte serum-free medium with added keratinocyte supplements, including bovine pituitary extract (BPE), human recombinant epidermal growth factor (EGF), fetal bovine serum (FBS), Dulbecco’s modified Eagle’s medium (DMEM), and 4′,6-diamidino-2-phenylindole (DAPI), were purchased from Gibco, Thermo Fisher Scientific (Waltham, MA, USA). Dimethyl sulfoxide (DMSO) and trypsin were purchased from Sigma-Aldrich (Saint Louis, MO, USA).

### 4.2. Preparation of Chitosan–Metal Oxide Films

First, 50 mg of chitosan was dissolved in 4 mL of 1% (*v*/*v*) acetic acid solution. A given mass of the metal precursor (zinc acetate) with an NH2:M molar ratio of (2:1; 5:1; 10:1) was added to the abovementioned solution. The mixture was then stirred for 1 h at room temperature to obtain a homogeneous dispersion, and the resulting solution was cast onto a clean Petri dish for 24 h until the total evaporation of the solvent. Data from the SEM, EDX, and FTIR analysis ascertaining the presence of these metal oxides inside are available in our previous work [17,26]. The chemical composition of the chitosan–metal oxide films is presented in Table 1.

### 4.3. Preparations of Graphene Oxide Fillers

Graphene oxide (GO) was obtained from graphite flakes using a modified Hummers’ method [54]. rGO was prepared according to the literature procedures [18,55]. The chemical composition of the chitosan–graphene films is presented in Table 2.

### 4.4. Preparation of Chitosan–Graphene Oxide Films

The procedure used to prepare CS:GO and CS:rGO films is similar to a previous study [18]. Briefly, 50 mg of chitosan was dissolved in 4 mL of 1% (*v*/*v*) acetic acid solution and kept under vigorous stirring for 2 h. Then, 1.5 mg of GO or rGO was dispersed in 2 mL of the 1% (*v*/*v*) acetic acid solution and submitted to sonication for 1 h 30 min. The suspension was gradually added to the chitosan solution, and the resulting mixture was stirred for an additional 1 h 30 min. The resulting solution was finally poured into plastic Petri dishes and dried at room temperature to form films.

### 4.5. Determination of Antifungal Activity

*Aspergillus flavus* ATCC 9643 and *Penicillium expansum* DSM 1282 were obtained from the American Type Culture Collection (ATCC) (Wesel, Germany) and DSMZ-German Collection of Microorganisms and Cell Cultures GmbH (Leibniz Institute, Germany), respectively. The selected fungal strains were seeded on Sabouraud slants, incubated for 120 h, and then used to prepare a spore suspension, the density/concentration of which was approximately 5 × 10^6^ CFU/mL. The obtained inoculum was transferred to the studied chitosan nanocomposites (2 cm × 2 cm), and covered with sterile film. Next, the samples were placed in a humid chamber and incubated in the dark for 48 h at 28 °C. At the same time, negative controls were prepared using native chitosan and commercial polyethene food foil. After the indicated time, microscopic observations of each tested sample were carried out.

### 4.6. Evaluation of the Antifungal Action Mechanisms of Chitosan Films

**Figure 6:**
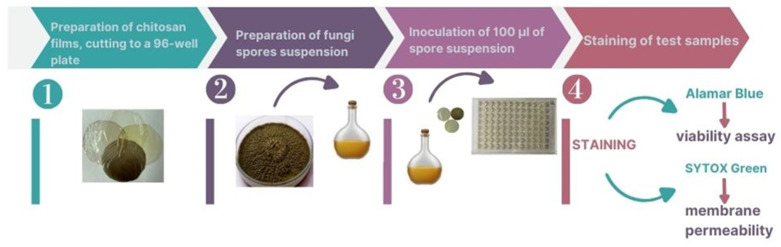
Alamar Blue or SYTOX Green test procedure for detecting mechanisms of antifungal action of chitosan films.

The antifungal activity mechanism of the modified and unmodified chitosan films was assessed on 96-well plates (Figure 6). The chitosan films were cut to the size of the bottom of the wells, and 100 µL of spore suspension (5 × 10^6^ CFU/mL) in Sabouraud’s medium was placed on the prepared films. The microtiter plates were incubated for 48 h at 28 °C. After incubation, the samples were stained with Alamar Blue (for 4 h) or SYTOX Green (for 5 min) dyes and read spectrophotometrically (at λ = 540 nm) with a microplate reader BMG 139 LabTech FLUOscar Omega (BMG LABTECH GmbH, Ortenberg, Germany) and MARS data analysis software (BMG LABTECH GmbH, Ortenberg, Germany, software version 5.10 R2, no 415-2045).

#### 4.6.1. Fungal Spore Viability Assays—Alamar Blue Test

Fungal samples on the selected chitosan films were prepared as described in Section 4.6. Alamar Blue was added to each well with 48 h fungal cultures and then incubated for 4 h at 28 °C. The reduction in Alamar Blue^®^ is shown as a percentage of the control—pure chitosan film—after fluorescence measurements at λ = 540 nm.

The Alamar Blue test involved measuring fluorescence resulting from the conversion of non-fluorescent resazurin to highly fluorescent resorufin. Resazurin, the active ingredient in Alamar Blue, can only be reduced to resorufin by living microorganisms.

#### 4.6.2. Estimation of Fungal Membrane Permeability

Fungal cultures on the selected chitosan films were prepared as described in Section 4.6. Sytox Green was added to each well with 48 h fungal cultures and then incubated for 5 min at 28 °C in the dark. Sytox Green Nucleic Acid Stain penetrates through damaged cell membranes. The fluorescence of fungal biomass was measured on a FLUOscar OMEGA (version 5.10) at an excitation wavelength of 485 nm and an emission wavelength of 535 nm. The obtained results are expressed as the percentage of fluorescence of the biotic control.

### 4.7. Genotoxicity

The comet assay was carried out under alkaline conditions, specifically following the protocol established by Singh et al. (1988) [49] with the modifications described by Blasiak and Kowalik (2000) [50]. A freshly prepared suspension containing 5 × 10^4^ cells/mL in a 0. 75% LMP agarose solution in PBS was smoothly spread over microscope slides that were pre-coated with 0. 5% NMP agarose. The cells were lysed at 4 °C for 1 h in a buffer solution containing 2.5 M NaCl, 100 mM EDTA, 1% Triton X-100, 10 mM Tris, and pH 10. Following the lysis process, the slides were immersed in an electrophoretic buffer composed of 300 mM NaOH and 1 mM EDTA, with a pH level exceeding 13, for a duration of 20 min to facilitate the unwinding of DNA strands. The electrophoresis experiment was carried out in an identical buffer solution under controlled conditions at a temperature of 4 °C for a duration of 20 min, applying an electric field strength of 0. 73 V/cm (28 mA). The slides were cleaned with water, drained, and then stained with a 2 mg/mL DAPI solution before being covered with cover slips. In order to avoid further DNA damage, the aforementioned steps were carried out in dim lighting conditions or in darkness. Every trial performed in the alkaline version of the comet assay featured a positive control consisting of two cell lines treated with H_2_O_2_ at a concentration of 20 mM for 10 min at 4 °C. The comets were observed at 200 magnification using an Eclipse fluorescence microscope (Nikon, Tokyo, Japan) attached to a COHU 4910 video camera (Cohu, San Diego, CA, USA) equipped with a UV-1 filter block (an excitation filter of 359 nm and a barrier filter of 461 nm) that was connected to a personal computer-based image analysis system (LuciaComet v. 6.0 (Laboratory Imaging, Praha, Czech Republic)). Fifty images were randomly selected from each sample.

## 5. Conclusions

The escalating concerns over plastic pollution and environmental sustainability have driven the search for alternative packaging materials that are biodegradable, renewable, and eco-friendly. Chitosan, derived from the abundant marine resource chitin, has attracted considerable attention as a sustainable alternative to conventional plastic packaging. Chitosan films offer several advantages, including antimicrobial properties, enhanced barrier performance, and compatibility with food products, positioning them as promising candidates for future packaging alternatives. Modified chitosan films show promising antifungal activity against *A. flavus* and *P. expansum*, which may suggest the potential of these materials as environmentally friendly alternatives to plastic packaging materials. However, more in-depth studies are needed to assess their long-term efficacy and safety in various practical applications.

## Figures and Tables

**Table 1:** Chemical composition of chitosan–metal oxide films.

Sample Code	Metal Precursor	NH_2_:Metal Precursor Molar Ratio
CS:ZnO 2:1	Zinc acetate	2:1
CS:ZnO 5:1	Zinc acetate	5:1
CS:ZnO 10:1	Zinc acetate	10:1

**Table 2:** Chemical composition of chitosan–graphene films.

Sample Code	Functionalized Graphene Filler
CS:GO	GO (3 wt%)
CS:rGO	rGO (3 wt%)

## Data Availability

The raw data supporting the conclusion of this article will be made available by the authors upon request.
